# Intraoperative superb microvascular ultrasound imaging in glioma: novel quantitative analysis correlates with tumour grade

**DOI:** 10.1007/s00701-025-06535-2

**Published:** 2025-05-06

**Authors:** Luke Dixon, Alistair Weld, Dolin Bhagawati, Neekhil Patel, Stamatia Giannarou, Matthew Grech-Sollars, Adrian Lim, Sophie Camp

**Affiliations:** 1 Division of Surgery and Cancer, Imperial College London, London, UK; 2 Radiology, Imperial College NHS Trust, London, UK; 3 Hamlyn Centre for Robotic Surgery, Department of Surgery and Cancer, Imperial College London, Exhibition Rd, London, SW7 2AZ UK; 4 Neurosurgery, Imperial College NHS Trust, London, UK; 5 Department of Computer Science, University College London, London, UK; 6 Lysholm Department of Neuroradiology, National Hospital for Neurology and Neurosurgery, University College London Hospitals NHS Foundation Trust, London, UK

**Keywords:** SMI, Ultrasound, Glioma, Microvascular

## Abstract

**Background:**

Accurate grading of gliomas is critical to guide therapy and predict prognosis. The presence of microvascular proliferation is a hallmark feature of high grade gliomas which to directly visualise traditionally requires targeted surgical biopsy of representative tissue. Superb microvascular imaging (SMI) is a novel high resolution Doppler ultrasound technique which can uniquely define the microvascular architecture of whole tumours.

**Methods:**

We examined both qualitative and quantitative vascular features of 32 gliomas captured with SMI, analysing flow signal density, vessel number, branching points, curvature, vessel angle deviation, fractal dimension, and entropy.

**Results:**

High-grade gliomas exhibit significantly greater vascular complexity and disorganisation, with increased fractal dimension and entropy, correlating with known histopathological markers of aggressive angiogenesis. The integrated ROC model achieved high accuracy (AUC = 0.95).

**Conclusions:**

This study leveraged SMI to provide further insights into the microvascular architecture of gliomas which is not resolvable by magnetic resonance imaging. Applying novel quantitative analysis the study demonstrated that there are quantifiable differences in vascular morphology between high grade and low-grade gliomas. This unique *in vivo* imaging of glioma vascularity and quantification warrants further exploration as a potential new diagnostic and prognostic tool that may support glioma management, intraoperative decision-making and informing future prognosis.

## Introduction

Discriminating the type and grade of glioma is imperative to guide treatment and predict prognosis. Although in recent years molecular features have taken a prominent role in stratifying gliomas, the presence of microvascular proliferation remains a pathological cornerstone for discriminating high-grade gliomas (HGG) from low-grade gliomas (LGG). Aberrant blood vessel growth is a defining feature of the highest grade, namely glioblastomas and grade 4 IDH mutant astrocytomas, and is characterised by multilayered small-calibre blood vessels. [22] Microvascular proliferation is driven by several mechanisms, including peri-necrotic hypoxia promoted vascular epidermal growth factor expression. [8] Presently, the detection of microvascular proliferation is limited to histopathological assessment of biopsied tissue. This requires the sampled tissue to be representative, with a risk of underestimating grade if an area of tumour without microvascular proliferation is biopsied.

Advanced ultrasound (US) microvascular Doppler techniques, such as superb microvascular imaging (SMI), have recently been developed and translated into clinical practice. SMI is a contrast-free, Doppler ultrasound technique developed by Canon Medical Systems (Tokyo, Japan), which uses an adaptive algorithm to filter out motion artefacts from true blood flow signals. This allows the delineation of a broader range of blood flow signals at a high resolution [13]. Outside of the CNS, SMI has been shown to improve the discrimination of malignant tumours from benign in several organ systems, including the breast and thyroid [7, 31]. This is based on increased sensitivity to neovascularity, a common feature of many types of malignant tumours. There has been limited exploration of SMI and other similar advanced Doppler techniques in intraoperative brain tumour imaging [2, 16, 17]. Analysis of SMI images has also been mainly limited to qualitative evaluation of vascular morphology and measures of vessel density, as quantification of vascular shape and complexity is challenging [2, 7, 16]. This challenge is well recognized in other fields of microvascular imaging. In retinal microvascular photographs the application of novel mathematical measures of image complexity such as fractal dimension and entropy analysis plus automated techniques for measuring vascular branching points and curvature have been well established in differentiating and grading different pathological processes [5, 12, 30]. Recently some of these metrics have been translated and explored in a different type of high resolution microvascular doppler imaging developed in a research setting in other more accessible tissues such as the breast and choroid of the eye [1, 27].

In this exploratory study, we will use already clinically available SMI to generate a unique, intraoperative, whole tumour view of glioma microvascular architecture. We will then qualitatively assess and apply several novel quantitative methods to these SMI glioma images. Through this approach, we hope to explore whether SMI can detect microvascular proliferation and discriminate HGGs from LGGs.

## Materials and methods

### Ethics approval

The study had local ethical approval from Health Research Authority (HRA) and Health and Care Research Wales (HCRW) authorities (REC reference: 22/WA/0259). Patients were retrospectively recruited and the need for informed consent was waived. The study was performed in accordance with the relevant guidelines/regulations and performed in accordance with the Declaration of Helsinki.

### Study participants and inclusion and exclusion criteria

Patients with histologically confirmed adult-type diffuse glioma who underwent intraoperative ultrasound (ioUS) guided resection of their tumour at the Department of Neurosurgery, Charing Cross Hospital, Imperial College Healthcare NHS Trust (London, United Kingdom) were retrospectively included in this study between January 2020 and October 2024.

The inclusion criteria were: age>18 years; confirmed adult-type diffuse glioma based on histology; and the use of ioUS with SMI acquisitions. The exclusion criteria were: age<18 years; and absent or incomplete intraoperative US data.

### Intraoperative ultrasound protocol and SMI acquisition

Intraoperative ultrasound was performed with a latest generation Canon i900 Aplio US system with a high-frequency iDMS Micro-Convex probe (i8MCX1, imaging frequency 8-8MHz, frame rate 18 - 25 frames per second), provided by Canon Medical Systems (Otawara, Japan). Image acquisition was performed by a neuroradiologist and neurosurgeon experienced with ioUS and a set scanning protocol previously described by the authors was followed [11]. After craniotomy, before and after opening the dura, 2D B-mode US and SMI clips sweeping through the entire tumour were obtained in two orthogonal planes following optimisation of focal zone, depth and gain. SMI acquisitions were performed in dual screen mode with B mode and SMI displayed side by side to allow accurate discrimination of intratumoral versus surrounding cerebral vessels. Monochrome SMI was used due to its greater sensitivity versus colour SMI and is routinely used in our practice to assist with tumour detection and to identify important vascular structures. Saved US images and video clips were anonymised on the scanner prior to transferring onto a workstation for offline processing.

### Qualitative image analysis

Two methodologies were used to qualitatively classify the patterns of microvascular architecture. A semiquantitative grading system was used to classify the variation of blood vessel size observed within the tumor into four distinct grades: grade 0, indicating the absence of blood flow signals; grade 1, calibre matches normal parenchyma; grade 2, uniformly dilated vessels relative to normal parenchyma ; and grade 3, heterogenously varied calibre. The morphological characteristics of the vessels were also categorized into four patterns based on prior experience and influenced by similar work in classifying liver lesions on doppler: a, sparse, straight unidirectional, penetrating flow signal; b, absent central signal with normal appearing parenchymal vessels displaced around ; c, absent central signal with an irregular, tortuous, rim of vessels flow projecting to the hypovascular core; d, intrinsic, multidirectional, irregular, tortuous flow signal [15, 20, 29]. Patterns a and b were classified as hypovascular supply, while patterns c and d was classified as hypervascular supply. Two neurosurgeons both with over 5 years experience in intraoperative ultrasound performed the rating.

### Tumour vessel mask segmentation

**Fig. 1:**
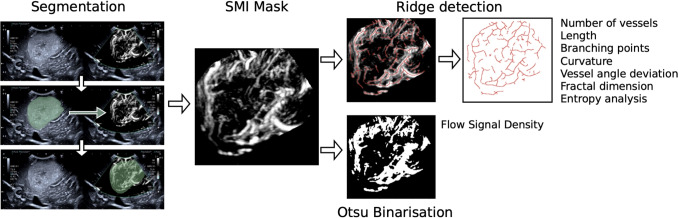
SMI Processing steps covering initial segmentation based on B-mode imaging on right then translated to mask the SMI image followed by ridge detection and Otsu binarisation of the masked SMI image

For quantitative analysis, SMI images were first converted to the Nearly Raw Raster Data format (Nrrd) and then reviewed on 3DSlicer [https://www.slicer.org/] where three representative SMI images of the tumours were manually chosen by a neuroradiologist (LD) blinded to final histology, with 10 years of experience in ultrasound and 5 years experience in neurosurgical ioUS. Images with the least artefacts (e.g., transient brain pulsation artefact) were selected. The intratumoral microvascular tree was then manually segmented by the same neuroradiologist to mask out surrounding normal cerebral and extra-cerebral vasculature using the side-by-side B-mode image and preoperative navigation MRI as references. Initial segmentations were performed on the B-mode imaging, which were then cloned and translated to overlay the SMI image using fixed coordinates. Once overlaid on the SMI image, the segmentation was refined to remove any overlaid graphical user interface elements burnt to the image by the ultrasound machine and constrained to the SMI window. Extracerebral vascular elements (e.g. sulcal vessels) were also excluded from the segmentation to limit the segmentation to purely intratumoral vessels (Fig. 1).

### Preprocessing of SMI masks

The SMI masks underwent two separate image processing steps to quantify both line-based and pixel-level features: 1) a simple noise robust, pixel intensity threshold for binarisation of the grayscale image, to assess flow signal density, and 2) vessel line detection and isolation for measurement of vascular properties such as curvature and fractal dimension.

The threshold binarisation was performed using Otsu’s automatic global thresholding method, a widely accepted technique for optimal threshold determining, to separate an image into a foreground (ones) and background (zeros) class via pixel intensity histogram analysis [23]. The method systematically evaluates a set of possible thresholds for separating the histogram into two distinct distributions, and the one which best maximizes the between-class variance and minimizes the within-class variance is chosen. For this work, the foreground is used to represent the flow signal and the background the low signal.

For the second method, the vessel lines were detected using the ridge detection plugin in Fiji (an ImageJ-based image processing software) [25]. This uses a well-established algorithm to identify curvilinear structures [26]. The algorithm detects 2D line points by applying Gaussian kernels and scale-space analysis to line profile models, enabling the detection of asymmetrical bar-shaped lines. These line points are linked into continuous lines and corresponding edge points along the normal direction are identified to accurately determine the true line positions; which are used for vessel measurement in this work. The following parameters for ridge detection were found to achieve optimal delineation of vessels line width 9.0, high contrast 230, low contrast 87, sigma 3.1, lower threshold 0.51 and upper threshold 1.53. From the plugin, each line is represented by a unique id and set of coordinates, and all lines belonging to an SMI image can then be saved in a CSV file.

### Quantitative image processing and feature extraction

The SMI images and corresponding binary maps and detected ridges were given a class label of HGG or LGG. The evaluation was performed using Python with the OpenCV, SciPy, Scikit-learn and SKimage libraries. Visualisation of the data was created using matplotlib and seaborn libraries.

For the binarised images, only flow signal density was measured. Flow signal density measures the ratio of white pixels (flow signal) to black pixels (background) within the segmented SMI region.

In the analysis of the detected ridges, the following seven characteristics were extracted: number of vessels, vessel length, curvature, number of branching points, vessel angle deviation, fractal dimension analysis, and entropy calculation. Number of vessels counted the number of vessels in each image. Vessel length measured the mean length of all lines in each image. Branching point counts the number of lines with more than two connections. Curvature measured the extent to which the lines changes direction along their paths, which is then averaged to reflect the overall tortuosity of the lines - a higher curvature suggests a more angled and irregular line and a lower curvature implies a straighter, less variable, line. The vessel angle deviation reflects the angular standard deviation of the mean resultant length which is a measure of the concentration of angles around a mean direction. This is based on the angles in radians, it sums up the sine and cosine of each angle and then calculates the magnitude of the resultant vector and normalizes it by dividing by the number of angles. The angular standard deviation uses the mean resultant length to calculate the standard deviation in angular data. A higher mean resultant length means angles are more concentrated around the mean direction, leading to a lower standard deviation. The purpose of the curvature and vessel angle deviation metrics is to measure the irregularity and degree of variation in the trajectories of the intratumoral vessels as increased disorganisation and variation of vessel orientations is well recognised in HGGs [18]. Vessel angle deviation has not been explored in SMI but this and related approaches looking at variance in vessel orientation have been applied in the analysis of retinal vasculature imaging and in cerebral vasculature on histological sampling [3, 21].

To quantify the structural complexity and overall organisation of the microvascular architecture, the fractal dimension and entropy of the ridges were measured. In fractal dimension analysis a box-counting method was used [24]. This approach divides the image into progressively smaller boxes and counts the number of boxes containing at least one white pixel. The slope of the logarithmic plot of the size of the box versus the count of the box approximates the fractal dimension, serving as a marker of structural complexity. The use of this novel quantification technique has been well explored in the analysis of other types of vascular imaging, such as in retinal vessel imaging, and has recently shown promise in the evaluation of similar microvascular Doppler imaging in other body systems such as the breast [12, 27]. Entropy calculation was performed using Shannon entropy, which is a mathematical measure of uncertainty or randomness in a system. In image analysis, it quantifies how complex the pixel intensities are in an image, assessing how varied or uniform the image is. High entropy suggests a more complex distribution of structures within an image whilst low entropy reflects a more uniform or predictable structure. Measures of entropy have been explored in other medical and biological imaging analyses, but their utility in assessing microvascular imaging is underexplored [19, 28]. An average of the final eight metrics across the three SMI images was then recorded for each glioma case.

### Statistical analysis

To assess the significance of each extracted microvascular metric for the HGG and LGG groups, normality was first tested using the Shapiro-Wilk test. Metrics that were normally distributed (p> 0.05) were evaluated with independent t-tests, while non-normally distributed metrics were analysed with the Mann-Whitney U test. The Pearson correlation matrix is then calculated to measure the metric interrelationships.

The potential of each metric for separating HGG and LGG was evaluated via receiver operating characteristic (ROC) analysis, with AUC values, optimal thresholds, and accuracies recorded. Then a linear regression model combined all metrics for an integrated ROC assessment of discriminative power.

Further analysis of the separability of the classes, using the investigated metrics, is conducted using Principal Component Analysis (PCA). To investigate the potential separability in PCA space, which is a popular technique for preparing data for machine learning algorithms such as support vector machines or K-nearest neighbour.

**Table 1:** Patient characteristics and tumour details for low grade gliomas (LGG) and high grade gliomas (HGG)

Characteristics	LGG	HGG
Total	10	22
Mean Age	37.67 ± 10.06	55.31 ± 14.6
Sex (N, %)		
Female	4 (12.5%)	9 (28%)
Male	6 (18.7%)	13 (40.6%)
Tumour Location (N, %)		
Frontal	8 (25%)	8 (25%)
Parietal	1 (3.1%)	6 (18.7%)
Temporal	1 (3.1%)	7 (21.9%)
Insula	-	1 (2.9%)
Tumour type		
IDH mutant Astrocytoma	7 (Grade 2)	4 (1 Grade 3, 3 Grade 4)
Oligodendroglioma	3 (Grade 2)	1 (Grade 3)
Glioblastoma IDH wild type	-	17 (Grade 4)
Microvascular Proliferation	0	18 (81.8%)

**Fig. 2:**
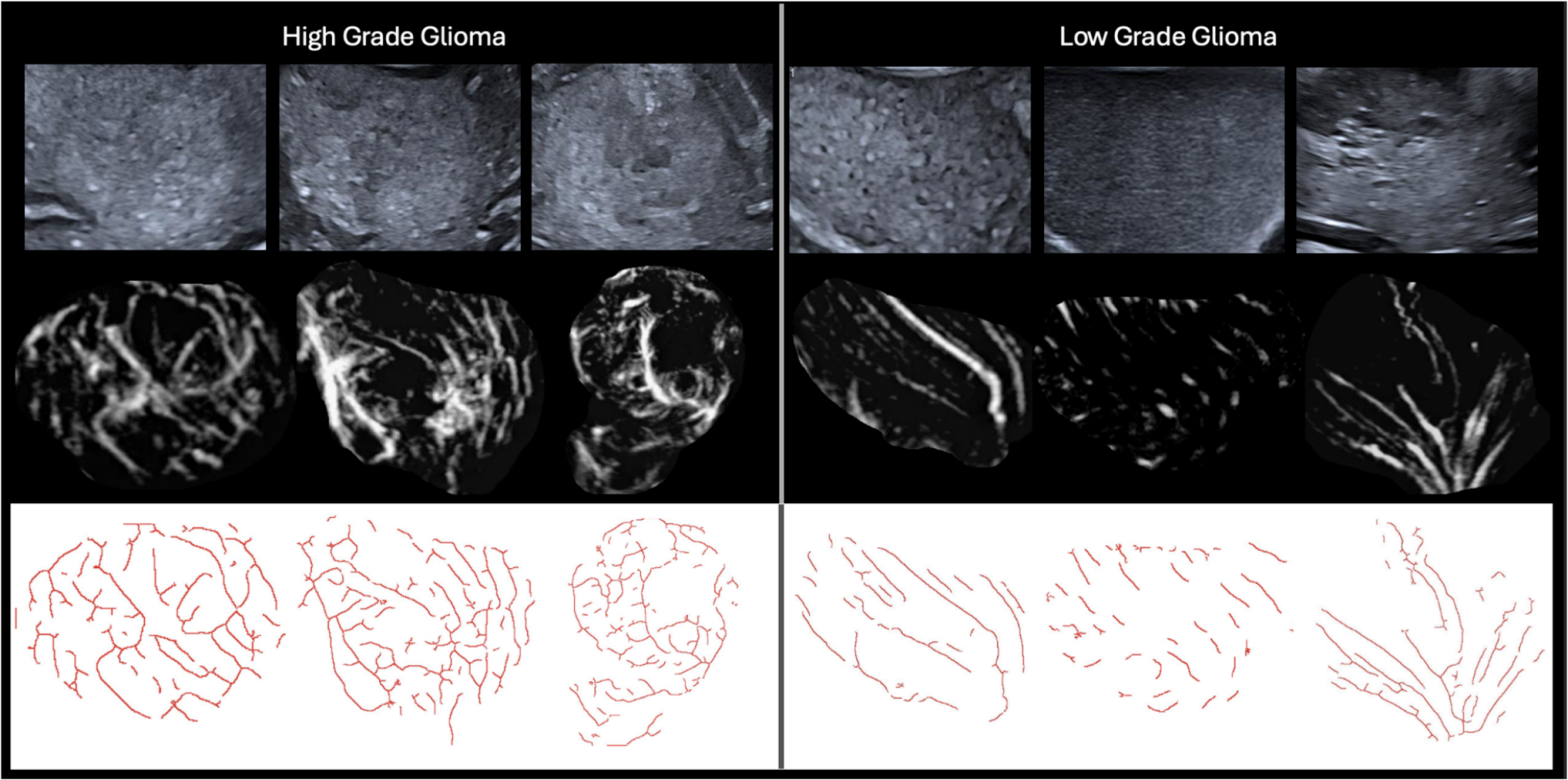
Examples of different HGGs (all IDH wild type grade 4 glioblastomas) and LGGs (all grade 2 IDH mutant astrocytomas) on B-mode (top row), SMI (middle row) and SMI following ridge detection (bottom row). Note the greater vascular density and more complex plus disorganised appearance to the microvascular architecture in the HGGs versus the more uniform and structured morphology of the vessels in the LGGs

## Results

### Population and clinical characteristics

A total of 32 patients (13 females and 19 males) who underwent ioUS guided resection of brain tumours at our hospital were retrospectively recruited in the study, 22 patients with HGG and 10 with LGG. The pathological diagnosis according to the current WHO 2021 classification in the HGG group was 17 grade 4 IDH wild type glioblastoma, 3 grade 4 IDH mutant astrocytomas, 1 grade 3 anaplastic IDH mutant astrocytoma and 1 grade 3 anaplastic oligodendroglioma whilst in the LGG group there was 7 grade 2 IDH mutant astrocytomas and 3 grade 2 oligodendrogliomas (clinical information summarised in Table 1). Microvascular proliferation was reported in 15 of the 17 glioblastomas (88.2%), 3 of the grade 4 IDH mutant astrocytomas (75%) and in the sole grade 3 oligodendroglioma. Characteristically no microvascular proliferation was noted in the grade 3 IDH mutant astrocytoma or the low grade glioma group.

**Table 2:** Qualitative assessment of microvascular architecture by two readers with interobserver agreement and significance testing of features against final histological outcome of LGG or HGG

	LGG		HGG			p-value	
	Reader 1	Reader 2	Reader 1	Reader 2	Kappa	Reader 1	Reader 2
Vessel Size					0.476	0.000*	0.004*
1. Normal	5	2	1	0			
2. Uniformly enlarged	3	6	1	4			
3. Variable	2	2	20	18			
Vessel Morphology					0.422	0.000*	0.005*
A) Linear, penetrating	9	5	1	1			
B) Absent with normal surrounding	0	1	3	1			
C) Irregular around a hypovascular core	0	3	7	11			
D) Intrinsic irregular, multidirectional	1	0	12	10			

* Denotes a significant difference (p<0.05) in the metric between the two groups

**Table 3:** Comparison of various quantitative microvascular metrics between HGG and LGG groups

Metric	HGG Mean (SD)	LGG Mean (SD)	p-value	AUC	95% CI	Threshold	Accuracy
Flow Signal Density	0.18 (0.09)	0.06 (0.04)	0.000*	0.92	(0.85 - 0.97)	0.07	0.88
Number of Vessels	177.36 (71.87)	100.37 (83.75)	0.000*	0.82	(0.70 - 0.91)	130.00	0.79
Length	158.41 (80.86)	149.19 (86.95)	0.535	0.54	(0.41 - 0.67)	121.20	0.60
Branching Points	64.09 (27.00)	32.50 (30.38)	0.000*	0.83	(0.71 - 0.92)	39.00	0.81
Curvature	0.57 (0.03)	0.55 (0.05)	0.004*	0.68	(0.55 - 0.79)	0.54	0.75
Vessel Angle Deviation	1.31 (0.14)	1.18 (0.19)	0.001*	0.71	(0.59 - 0.83)	1.17	0.77
Fractal Dimension	1.04 (0.03)	0.96 (0.03)	0.000*	0.93	(0.87 - 0.98)	1.00	0.90
Entropy	0.09 (0.03)	0.06 (0.03)	0.000*	0.77	(0.66 - 0.86)	0.07	0.71
Integrated	-	-	-	0.95	0.91 - 0.99	0.63	0.92

* Denotes a significant difference (p<0.05) in the metric between the two groups

**Fig. 3:**
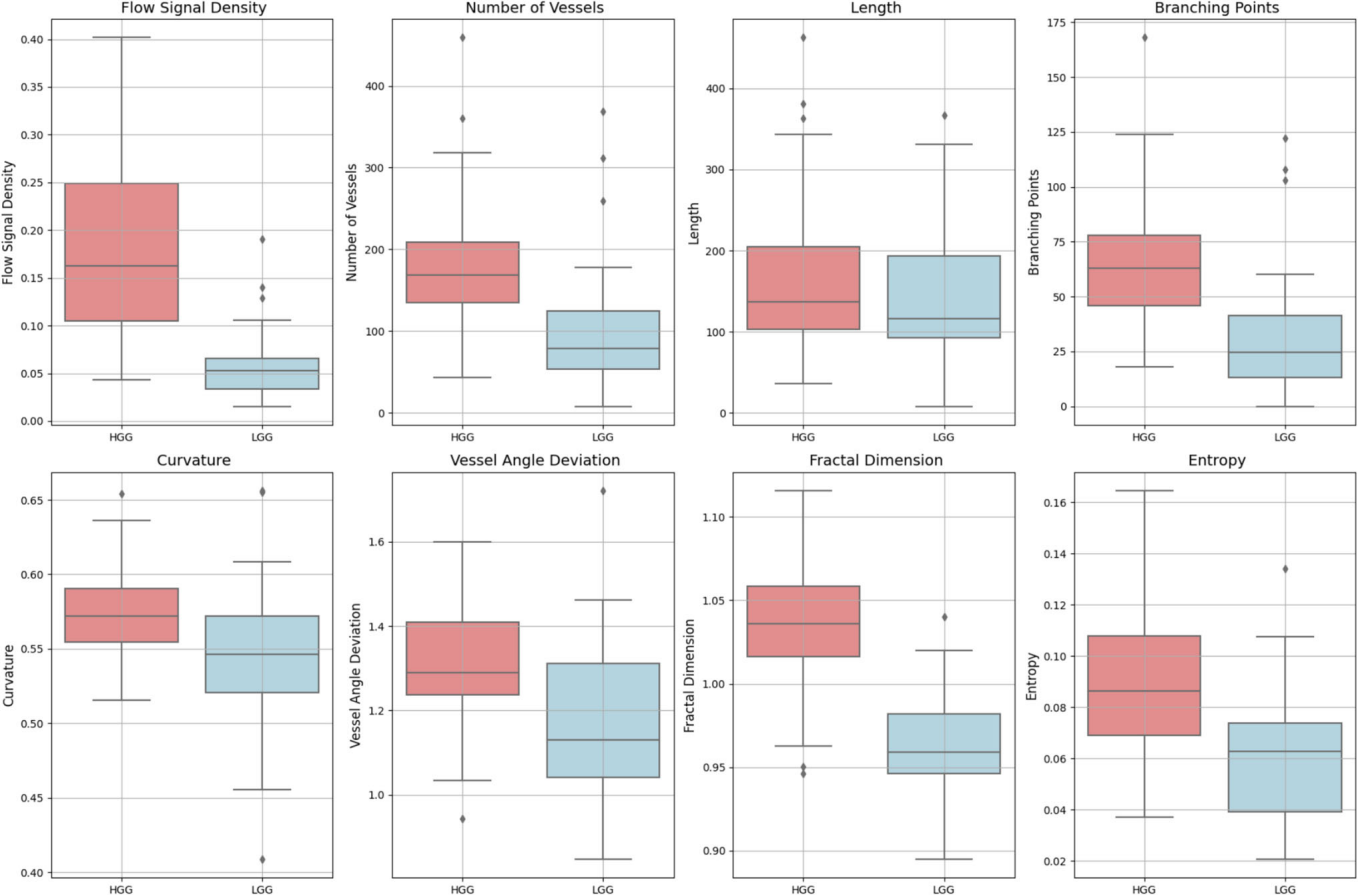
Boxplots of the distributions of the different quantitative microvascular metrics for HGG and LGG. Significant difference (p<0.05) between HGG and LGG was noted across all metrics except ’Length"

**Fig. 4:**
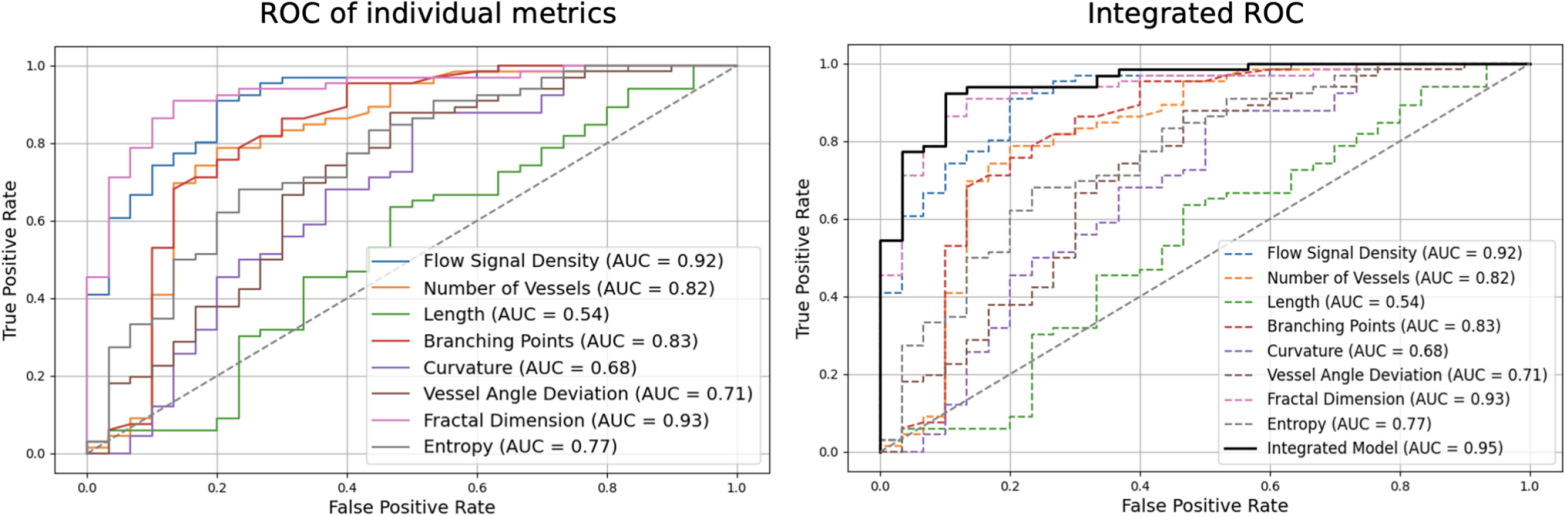
Summary of ROC of different quantitative microvascular metrics for differentiating HGG from LGG (right) plus integrated ROC of all metrics. Fractal dimension and flow signal density demonstrated the highest AUC whilst Length exhibited poor separation

### Qualitative features

The microvascular architecture was qualitatively different between the HGG and LGG groups in both vessel size and morphology (see Fig. 2 for example images of different gliomas and Table 2 summarizing the qualitiative scoring). In vessel size the majority of LGGs were classified as having either normal or uniformly enlarged vessels (8/10, 80%) whilst in the HGGs the majority were classified as heterogenously variable in size by both readers (18/22, 90%, 20/22, 90.9%). In vessel morphology the LGGs were predominantly classified as exhibiting pattern A) Linear, penetrating (9/10 90%, 5/10, 50%) whilst in HGGs the majority were classified as either pattern (C) Irregular around a hypovascular core (7/22, 31.8%, 12/22, 54.5%) or pattern (D) Intrinsic irregular, multidirectional (12/22, 54.5%, 10/22, 45.5%). When grouping vessel size 1 and 2 as organised and 3 as disorganised there was a significant difference between both LGG and HGG groups and similarly when classifying morphology patterns (A) and (B) as hypovascular and (C) and (D) as hypervascular there was a significant difference between HGGs and LGGs. Cohen’s Kappa showed moderate agreement between both readers for both vessel size (0.476) and morphology pattern (0.422).

### Quantitative features

**Fig. 5:**
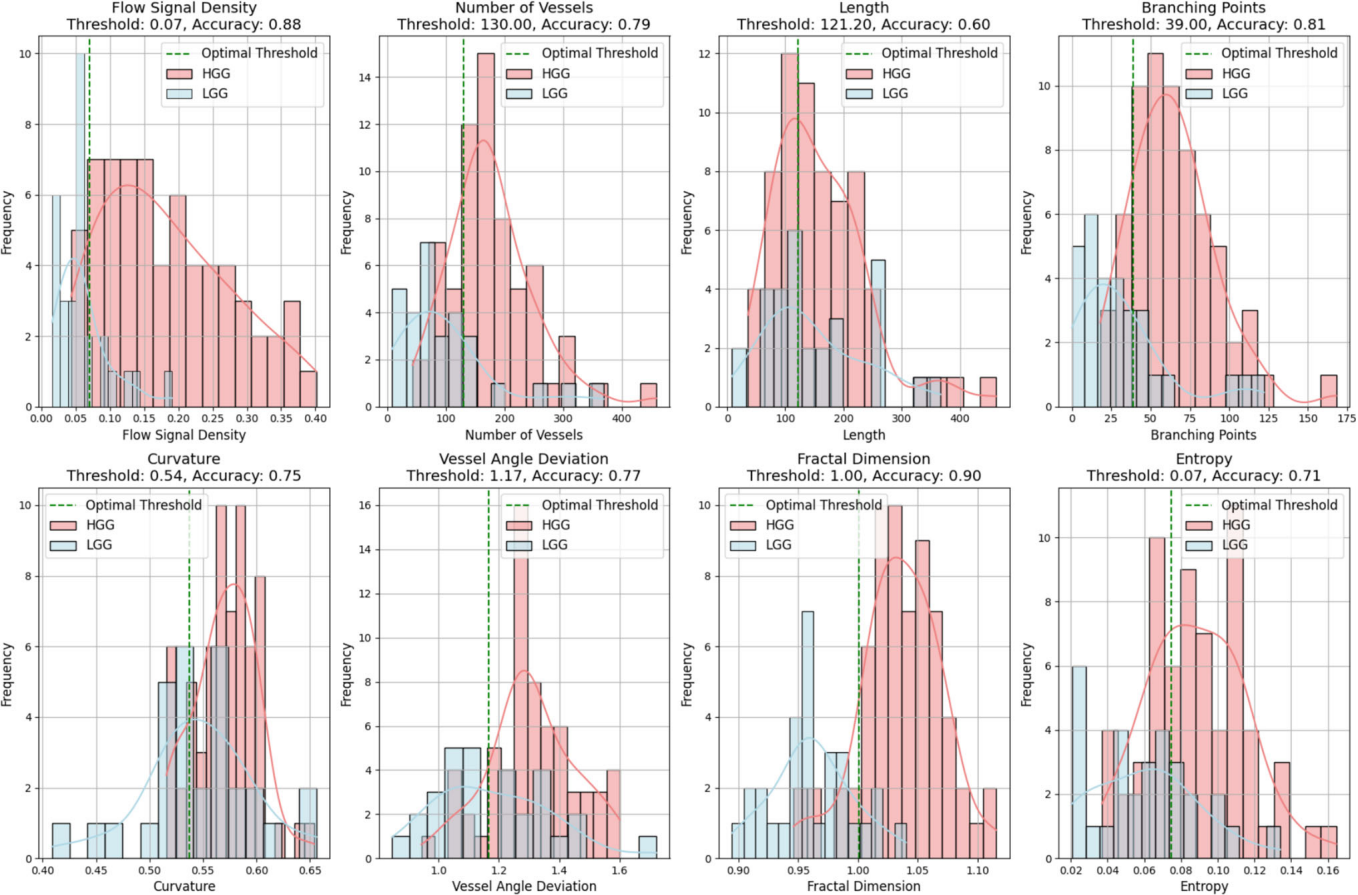
Histograms of the distributions of the different quantitative microvascular metrics for HGG and LGG with optimal threshold lines for discrimination derived from the ROC analysis

The results of the eight quantitative characteristics averaged between the HGG and LGG groups are summarised in Table 3. The distributions are visualised using the boxplots in Fig. 3. Seven of the eight metrics showed a significant difference between HGG and LGG. The flow signal density, number of vessels, branching points, curvature, vessel angle deviation, fractal dimension analysis, and entropy calculation were significantly higher in the HGG group while there was no significant difference in length. Fractal dimension analysis (AUC = 0.93, 95% CI: 0.87-0.98) and flow signal density (AUC = 0.92, 95% CI: 0.85-0.97) demonstrated the highest classification potential, achieving high accuracy at optimal thresholds (0.90 and 0.88, respectively) with significant p-values (p< 0.001). Branching points (AUC = 0.83 CI: 0.71-0.92) and number of vessels (AUC = 0.82 CI: 0.70-0.91) also showed a strong discriminative power, while entropy calculation, vessel angle deviation and curvature exhibited moderate classification capability, with AUC values of 0.77, 0.71, 0.68, respectively, and significant p-values (p< 0.01). Length displayed limited ability to distinguish HGG from LGG (AUC = 0.54, p = 0.535). A linear regression model integrating all features achieved the highest classification potential with an AUC of 0.95. Figure 4 summarises the ROCs of the individual and pooled integated metrics and in Fig. 5 histograms of the distributions with the optimal threshold lines from the ROC analysis are presented.

**Fig. 6:**
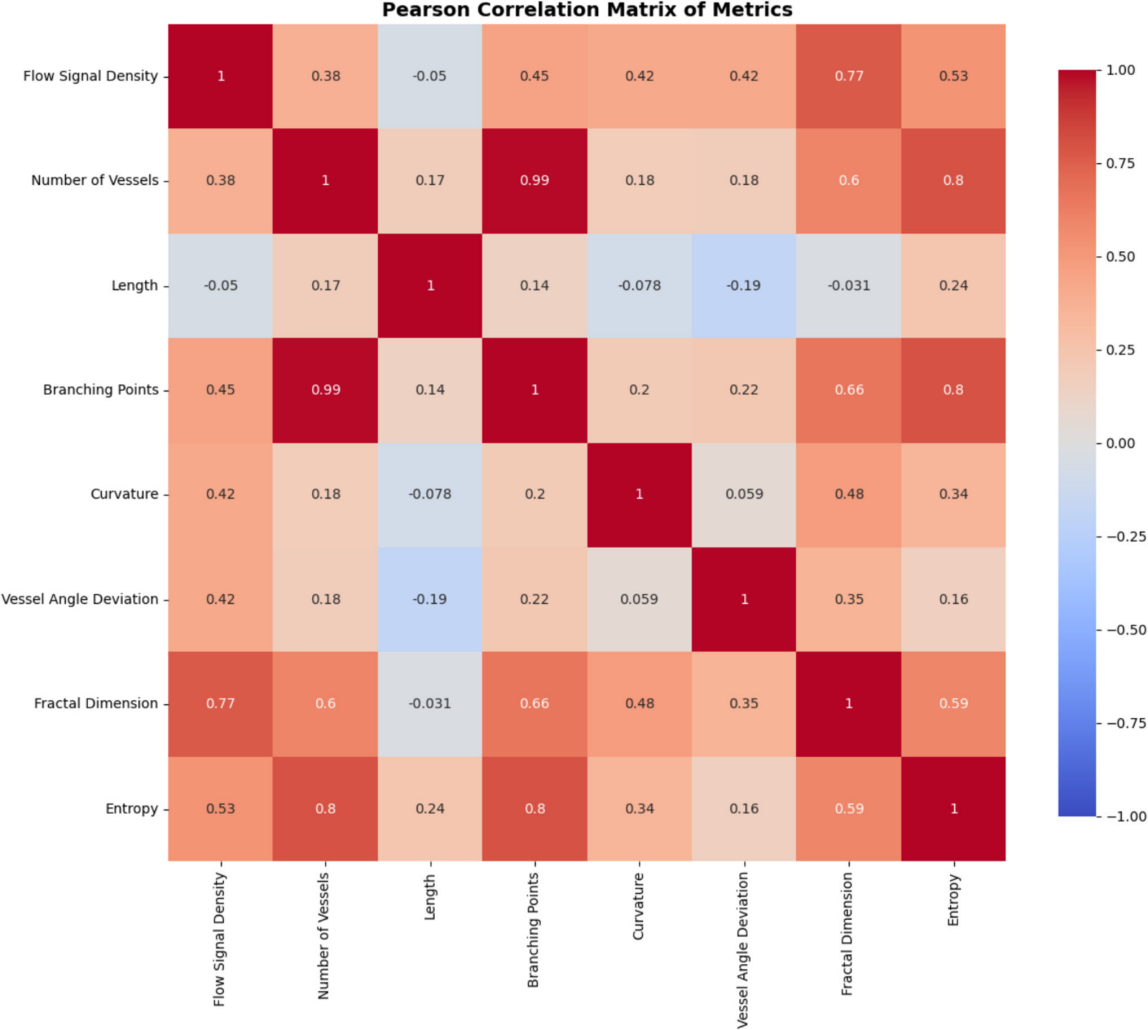
Pearson correlation matrix of microvascular quantitative metrics. Red cells reflect positive correlation and blue cells negative correlation. The majority of the metrics exhibited intermediate to moderation intercorrelation with only Branching points and Number of vessels showing almost equal correlation

A Pearson correlation matrix measuring the interrelationship of each microvascular metric is shown in Fig. 6. This found a very strong positive correlation with branching points and number of vessels (0.99). Strong correlations of entropy calculation with number of vessels (0.8) and branching points (0.8) plus fractal dimension analysis and flow signal density (0.77) and BP (0.66) were also observed. The length was weakly correlated with all other characteristics.

**Fig. 7:**
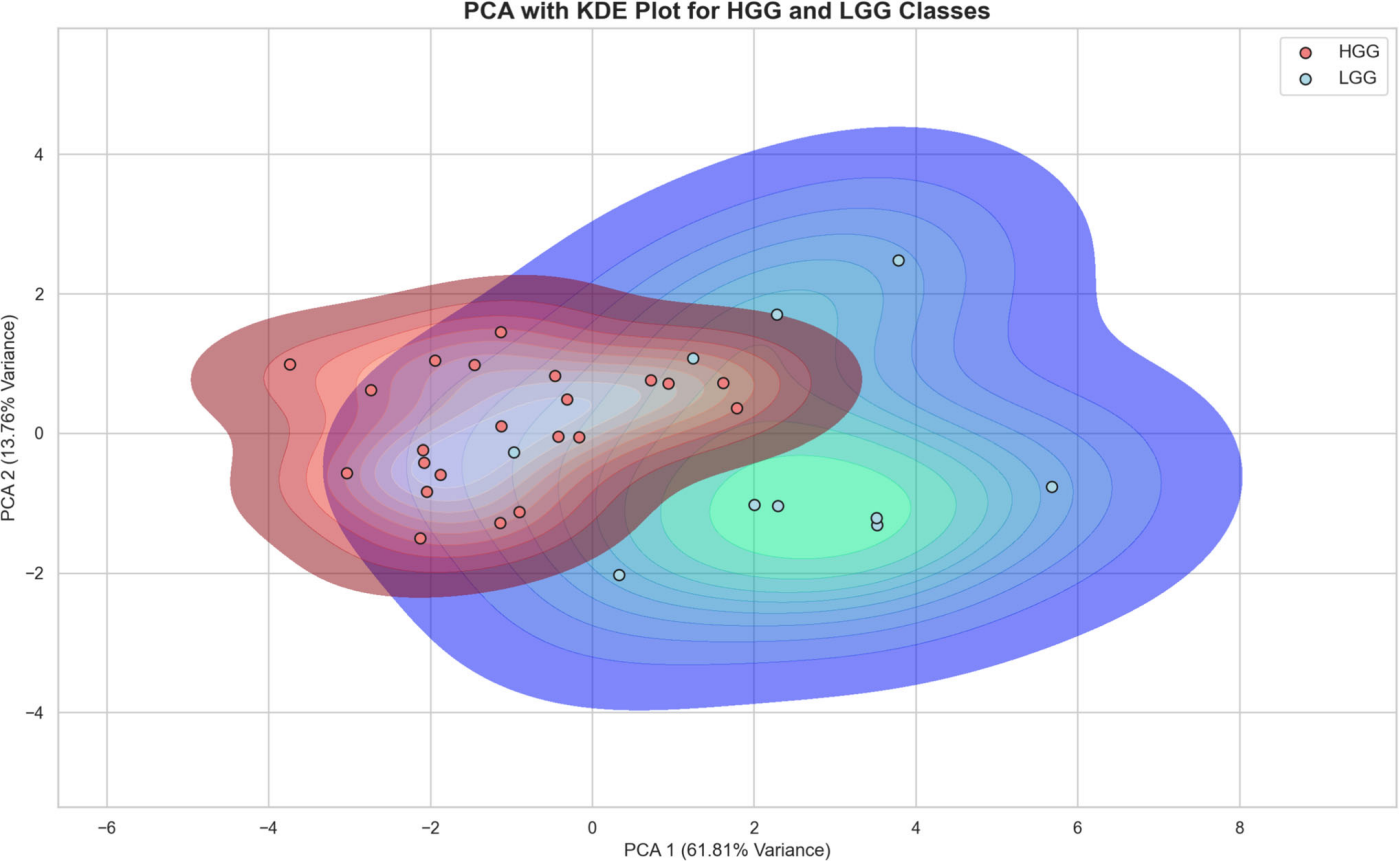
Principal component analysis of the microvascular metrics across the HGG and LGG groups with kernel density estimation (KDE) plot to visualise the underlying distribution of data across reduced dimensions. The plot shows overlap of the two classes but appreciable clustering of both groups suggesting that there are distinct features that with larger data sets may facilitate machine learning classification

To complete the exploratory analysis and to further measure the separation of the microvascular metrics, the 2D PCA of the HGG and LGG groups is shown in Fig. 7. For visualisation, Kernel Density Estimation (KDE) is applied onto the 2D PCA plot, to show the overlap of the two groups. This visualisation suggests that there are shared microvascular features between the two classes, there is however appreciable clustering with the HGG group which appears more compact possibly reflecting more consistent features compared to the more dispersed LGG group. The explained variance for the two components is shown on the axes in the figure. PCA Component 1 explains 61.8% of the variance, and Component 2 explains 13.76%. Together, these two components account for 75.56% of the total variance, which is a strong representation of the data. This result suggests that the PCA projection and the first two components may be effective for machine learning classification of HGG and LGG.

## Discussion

In this exploratory study, we used a clinically available, non-contrast, high-resolution microvascular Doppler ultrasound technique called SMI to generate a whole-tumour view of microvascular architecture in gliomas. This allowed dynamic visualisation of the tumour vascular network, surpassing the spatial and temporal resolution of conventional modalities such as CT and MRI. We demonstrated that both qualitative scoring of microvascular characteristics and translation of conventional plus novel quantitative techniques can help discriminate tumour grade [5, 12, 27]. Fractal dimension analysis, branching points, flow signal density and number of vessels offered the highest discriminatory power with moderate separation also noted with measures of vessel curvature, angulation and entropy. Our observation that flow signal density and vessel number correlate with HGGs parallels the histological observation of increased neoangiogenesis and microvascular proliferation in HGGs [9, 14, 18]. The vessel number metric showed a very high inter-metric correlation with the number branching points which was almost equal (0.99), suggesting that these effectively measure the same feature of vessel number. Interestingly, there was a weaker correlation between flow signal density and vessel number, implying that these capture discrete features, likely secondary to the flow signal density being influenced by the calibre of vessels as well as the number of vessels. Of all the metrics, fractal dimension analysis showed the highest AUC. Fractal dimension analysis is a measure of structural complexity and irregularity which quantifies how much detail is present in a structure at different scales. In the context of gliomas, a high fractal dimension suggests a more complex and disordered vasculature which matches the pathological findings in HGG which are defined by the presence of heterogenous and irregular microvascular proliferation fueled by several factors including hypoxia-driven neoangiogenesis [9, 18]. Furthermore, our observation of significantly higher curvature and vessel angle deviation in HGGs highlights the increased vessel tortuosity and disorganized orientation within these tumors. These metrics align well with the irregular, chaotic neoangiogenesis that characterizes HGGs, where rapid tumor expansion outstrips the formation of a structured blood supply, leading to a disordered vascular architecture [9, 14, 18]. This is further shown by the higher entropy feature seen in the HGG group which is a measure of randomness and disorder over the whole intratumoral microvascular image.

A limitation of microvascular Doppler in brain tumours is its confinement to the intraoperative setting, as imaging through the skull is not feasible. Despite this, SMI and quantitative analysis hold several potential clinical applications. Firstly, it may assist in identifying the high-grade vascular components for resection and sampling, supported by early data on improved tumour boundary detection with SMI [4]. Secondly, prior studies have linked specific microvascular patterns with prognosis in HGGs, glomeruloid vascular proliferation and vascular mimicry for instance are both associated with worse progression free survival and overall survival [6]. Thus, SMI-based quantification could serve as a prognostic biomarker alongside histological and molecular data. Additionally, varying vascular patterns may respond differently to antiangiogenic therapy, offering a route to individualized treatment. Finally, while limited to the intraoperative setting, there is early work showing that ultrasound monitoring of brain tumours is potentially feasible through sonolucent cranioplasty plates that can be placed at the time of surgical resection, which would further expand the utility of microvascular Doppler in the postoperative monitoring setting [10].

This study has further limitations. (i) Our findings are based on a small cohort, limiting generalizability. (ii) Several intraoperative factors-such as operator skill, tumour depth, and imaging artifacts-could affect metric consistency. (iii) Manual segmentation introduces subjectivity and is time-intensive.

Larger, ideally multi-centre studies are required to further validate SMI’s potential in glioma characterisation. Expanding analysis across tumour types and correlating metrics with survival outcomes would help refine clinical utility. Although our sample was small, the high AUC of integrated metrics suggests potential for machine learning or deep learning approaches to further develop microvascular imaging biomarkers.

## Conclusion

This exploratory study underscores the potential of SMI, an already clinically available imaging technique, as a new tool for glioma grading, providing real-time, dynamic imaging of the tumour microvascular architecture. Quantitative analysis of SMI metrics, such as fractal dimension analysis and flow signal density, may provide an opportunity to define distinguishing features that further refine the classification of glioma.

## Data Availability

The datasets used and/or analysed during the current study are available from the corresponding author on reasonable request.
